# Overexpression of p16^INK4a^ Serves as Prognostic Marker in Squamous Cell Vulvar Cancer Patients Treated With Radiotherapy Irrespective of HPV-Status

**DOI:** 10.3389/fonc.2019.00891

**Published:** 2019-09-11

**Authors:** Nathalie Arians, Elena-Sophie Prigge, Tereza Nachtigall, Miriam Reuschenbach, Stefan Alexander Koerber, Juergen Debus, Magnus von Knebel Doeberitz, Katja Lindel

**Affiliations:** ^1^Department of Radiation Oncology, Heidelberg University Hospital, Heidelberg, Germany; ^2^Heidelberg Institute of Radiation Oncology (HIRO), Heidelberg, Germany; ^3^National Center for Tumor Diseases (NCT), Heidelberg, Germany; ^4^Department of Applied Tumour Biology, Institute of Pathology, Heidelberg University Hospital, Heidelberg, Germany; ^5^Clinical Cooperation Unit Applied Tumour Biology, German Cancer Research Centre (DKFZ), Heidelberg, Germany; ^6^Department of Gynecology and Obstetrics, Heidelberg University Hospital, Heidelberg, Germany; ^7^Clinical Cooperation Unit Radiation Oncology, German Cancer Research Center (DKFZ), Heidelberg, Germany; ^8^Department of Radiation Oncology, Heidelberg Ion-Beam Therapy Center (HIT), Heidelberg University Hospital, Heidelberg, Germany; ^9^German Cancer Consortium (DKTK), Partner Site Heidelberg, Heidelberg, Germany; ^10^Department of Radiation Oncology, Municipal Hospital Karlsruhe gGmbH, Karlsruhe, Germany

**Keywords:** vulvar squamous cell carcinoma, human papillomavirus, p16^INK4a^, radiotherapy, prognostic factors

## Abstract

**Purpose:** We aimed to evaluate the impact of HPV-driven carcinogenesis on outcome in vulvar squamous cell carcinoma patients (VSCC) treated with radiotherapy.

**Methods and Materials:** Analysis of clinical, pathological, and treatment data, HPV DNA-detection and -genotyping as well as p16^INK4a^ immunohistochemistry were performed in 75 VSCC patients. Kaplan–Meier-method was used to estimate locoregional control (LC), Progression-free survival (PFS), and Overall Survival (OS). Univariate survival time comparisons were performed using the log-rank-test. Chi-square/Fisher exact test was used to assess correlations between HPV DNA and p16^INK4a^ data, pathological, clinical, and treatment characteristics.

**Results:** 23/75 (30.67%) of all women had locoregional relapse, 7/75 (9.3%) systemic recurrence, and 35/75 (46.67%) died after a median follow-up of 26.4 months. 21.3% of the tumors were HPV DNA-positive, mostly (93.75%) for the high-risk (HR) HPV type 16. 25.3% showed p16^INK4a^-overexpression. 17.3% showed concomitant HPV DNA- and p16^INK4a^-positivity (cHPPVC). Patients with p16^INK4a^-overexpression, irrespective of the HPV DNA status, showed significantly better PFS (5-year-PFS 69.3 vs. 39.2%, *p* = 0.045), LC (5-year-LC 86.7 vs. 56.7%, *p* = 0.033) and a strong trend for better OS (5-year-OS 75.6 vs. 43.9%, *p* = 0.077). Patients with cHPPVC showed a trend for better PFS (5-year-PFS 72.7 vs. 41.3%, *p* = 0.082) and OS (5-year-OS 81.1 vs. 45.7%, *p* = 0.084) but no significant benefit for LC.

**Conclusions:** Patients with cHPPVC, indicating an etiological relevance of HPV in the respective tumors, showed a better, albeit not significant, prognosis. The sole detection of p16^INK4a^-overexpression is a prognostic factor for survival in vulvar cancer and indicates better prognosis after radiotherapy, independent of detection of HPV DNA. p16^INK4a^ should be used as surrogate marker for HPV-driven carcinogenesis in vulvar cancer with caution.

## Introduction

Vulvar cancer represents only 3–5% of all gynecologic malignancies and prospective data regarding prognostic factors, outcome, and the role of HPV are rare. Two major etiologies have been described: HPV infection and chronic inflammatory dermatosis or autoimmune conditions. Histologically, precursor lesions of non-HPV-related vulvar carcinomas are differentiated vulvar intraepithelial neoplasia (VIN) whereas HPV-induced carcinomas arise from the usual type VIN and are of basaloid or warty type. HPV DNA is detected in >80% of all VIN lesions while HPV prevalence amongst invasive vulvar carcinomas seems to be lower. A recent publication (1) revealed that HPV contribution in invasive vulvar carcinoma worldwide has probably been overestimated: only 25.1% of 1,709 tumors from 39 countries were HPV-driven, as indicated by the simultaneous detection of HPV DNA and p16^INK4a^-overexpression. Among these, high-risk HPV 16 was the most common genotype (72.5%), followed by HPV 33 (6.5%) and HPV 18 (4.6%).

In HPV-transformed cells, expression of the viral oncogenes E6 and E7 leads to degradation and inactivation of the tumor suppressor proteins p53 and pRb, resulting in cell cycle dysregulation and uncontrolled cellular proliferation (2–4). E7 oncogene signaling further results in extensive overexpression of the cell cycle regulator p16^INK4a^, which is triggered by the removal of repressive trimethyl marks in the promoter region of the p16^INK4a^-encoding gene CDKN2A via the KDM6B demethylase (5). Since HPV DNA OR p16^INK4a^-overexpression may occur individually in different biological contexts, it is essential to assess the presence of both markers when determining a functional relevance of HPV in carcinogenesis (6). Only tumors, in which both—HR-HPV DNA AND diffuse p16^INK4a^-expression—are found, can biologically be considered as HPV-induced/HPV-driven (7).

The prognostic significance of the tumoral HPV status and the use of immunohistochemical p16^INK4a^-overexpression as a surrogate marker of HPV-induced transformation in vulvar squamous cell carcinoma (VSCC) are discussed controversially. Other squamous cell carcinomas, especially of the anogenital or head and neck region, are well-known to be associated with high-risk HPV. Furthermore, overexpression of the cell cycle regulator protein p16^INK4a^ correlates with the presence of HPV DNA in cervical, anal or oropharyngeal cancer and p16^INK4a^-overexpression has been found to be of independent prognostic value for the response to radiation treatment (6, 8–13). A recent study revealed substantial mismatch between p16^INK4a^-overexpression and HPV DNA detection in VSCC and evidence arises that p16^INK4a^ itself might function as an independent prognostic marker in vulvar cancer patients irrespective of an association with HPV (14, 15).

The study aimed to evaluate the association of HPV-driven carcinogenesis, indicated by HPV DNA and p16^INK4a^-overexpression, with clinical outcome in patients with VSCC treated with radiotherapy. In addition, the prognostic value of both markers was assessed individually in this cohort.

## Materials and Methods

### Patient Selection and Data Collection

Data from 119 patients with histologically proven vulvar squamous cell carcinoma who were treated with curatively intended radio- or radiochemotherapy at the Department of Radiation Oncology at University Hospital Heidelberg from 01/1999 to 04/2014 were retrieved from clinical databases. Tumor tissue biopsies from 92 patients taken at the time of primary diagnosis and/or time of disease recurrence were available for HPV DNA and p16^INK4a^ analyses. The biopsies were obtained as formalin-fixed, paraffin-embedded tissue from local pathologists and the Institute of Pathology of University Hospital Heidelberg. For statistical analyses, only patients with tumor tissue samples and thus available HPV DNA- and p16^INK4a^-status from time of radiotherapy were included in the current analysis. Patient characteristics, tumor stage, details of oncological treatment including radiotherapy admission and follow-up exams were obtained from medical records. After exclusion of patients with insufficient clinical information, follow-up data or missing tumor tissue biopsies, 75 patients were included in the current analysis. Pathological and treatment characteristics are summarized in Tables 1, 2.

**Table 1:** Clinical and pathological characteristics.

	**Entire cohort n(75)**	**cHPPVC n(13)/17.3%**	**Non-cHPPVC n(62)/82.67%**	***p*-value**	**p16^**INK4a**^ positive n(19)/25.3%**	**p16^**INK4a**^ negative n(56)/74.67%**	***p*-value**
Date of first diagnosis				0.139			0.058
1991–1998	7	1	6		1	6	
1999–2006	22	1	21		2	20	
2007–2014	46	11	35		16	30	
T-status				0.001^*^			0.003^*^
T1	35	6	29		10	25	
T2	29	1	28		2	27	
T3	7	4	3		4	3	
T4	3	2	1		3	0	
N-status at time of fist diagnosis				0.564			0.420
N0	36	6	30		10	26	
N+	39	7	32		9	30	
ECS	10	2	6		2	6	
N-status at time of first recurrence				0.280			0.547
N0	44	8	36		11	33	
N+	22	2	20		5	17	
Grading				0.225			0.423
G1	5	0	5		0	5	
G2	46	6	40		11	3235	
G3	20	6	14		7	13	
≥ 8 mm	17	3	14		5	11	
p16^INK4a^-status				<0.001^*^			-
Positive	19	13	6		19	0	
Negative	56	0	56		0	56	
HPV-status				<0.001^*^			<0.001^*^
Positive	16	13	3		13	3	
Negative	59	0	59		6	53	
Survival data							
Deaths	35	3	32	0.056	5	30	0.035^*^
Locoregional recurrence after RT	23	2	21	0.163	2	21	0.023^*^
Distant metastases	7	0	7	0.248	1	6	0.428

* *p <0.05, statistically significant*.

**Table 2:** Treatment characteristics.

	**Entire cohort n(75)**	**cHPPVC n(13)/17.3%**	**non-cHPPVC n(62)/82.67%**	***p-*value**	**p16^**INK4a**^ positive n(19)/25.3%**	**p16^**INK4a**^ negative n(56)/74.67%**	***p*-value**
Surgical resection				0.319			0.0445
Yes	73	12	61		18	55	
No	2	1	1		1	1	
Inguinal LNE				0.511			0.205
Yes	54	10	44		12	42	
No	20	3	17		7	13	
Pelvic LNE				0.045^*^			0.165
Yes	9	4	5		4	5	
No	65	9	56		15	50	
R-status				0.291			0.516
R0	61	10	51		16	45	
R+	6	2	4		2	4	
Resection margins				0.588			0.551
<8 mm	35	6	29		9	26	
≥ 8 mm	14	2	12		4	10	
Radiotherapy (RT) setting				0.273			0.167
•Definitive RT	5	2	3		2	3	
•Neoadjuvant RT	3	1	2		2	1	
•Adjuvant RT	67	10	57		15	52	
Radiotherapy timing				0.006^*^			0.033^*^
RT as part of initial treatment	44	12	32		15	29	
RT as part of salvage treatment	31	1	30		4	27	
BED							
>70 Gy	18	4	14		4	14	
>60 Gy	55	8	47		12	43	
>50 Gy	72	13	59		19	53	

* *p <0.05, statistically significant*.

### HPV DNA Detection and Genotyping

DNA extraction was performed using QIAGEN DNeasy© Blood and Tissue Kit from the available, formalin-fixed and paraffin-embedded tissue sections. For polymerase chain reaction (PCR) consensus HPV primers-sets (modified GP5+6+) were used. Samples with bands of about 150 base pairs corresponding to the length of positive control amplicons were considered positive.

We performed genotyping with positive samples using the Multiplex HPV Genotyping Kit© from Multimetrix GmbH (Regensburg, Germany, now DiaMex, Heidelberg, Germany) for subtypes HPV6, HPV11, HPV16, HPV18, HPV26, HPV31, HPV33, HPV35, HPV39, HPV42, HPV43, HPV44, HPV45, HPV51, HPV52, HPV53, HPV56, HPV58, HPV59, HPV66, HPV68, HPV70, HPV73, and HPV82. Samples were considered positive if median intensity of fluorescence in the Luminex Analyzer was 10 or higher.

### p16^INK4a^ Immunohistochemistry

p16^INK4a^ immunohistochemistry was performed on 2 μm tissue sections using the CINtec p16^INK4a^ histology kit (mtm Laboratories, Heidelberg, Germany) according to the manufacturer's instructions. The p16^INK4a^ antigen was detected with a mouse monoclonal anti-human p16^INK4a^ antibody (E6H4™). Tissue sections with a diffuse (clonal) tumoral p16^INK4a^ staining as previously described (10) in more than 50% of tumor cells were considered positive. Lesions showing either focal staining only or no p16^INK4a^ expression at all were considered as p16^INK4a^-negative.

### Statistical Analysis

All survival end-points were calculated starting from the date of start of radiotherapy. Overall survival (OS) was then defined as time to death from any cause. Time to locally progressive disease of the primary tumor or regional lymph nodes was determined as locoregional control (LC). Progression-free survival (PFS) was defined as time to local/distant recurrence or death. Distant control (DC) was defined as time to distant metastases. All patients with no event at the last follow-up were censored. In detail, events were death for OS, locoregional progressive disease, distant metastases or death for PFS, local recurrence or recurrence in regional lymph nodes for LC, distant metastases for DC. Chi-square/Fisher exact test were used to assess correlations between staining results and pathological, clinical, and treatment characteristics. Differences between the p16^INK4a^-positive vs. the p16^INK4a^-negative group as well as the group with cHPPVC (concomitant HPV and P16^INK4a^ Positive Vulvar Cancer) vs. the rest of the patients were assessed using the chi square/Fisher exact test. The Kaplan–Meier method was used to estimate LC, PFS, DC, and OS for various group partitions. Univariate survival time comparisons were performed using the log-rank test. The statistical analysis was performed using SPSS version 24. The study was granted ethical approval by the local ethics committee.

## Results

### Patients' and Treatment Characteristics (Tables 1, 2)

At the time of primary diagnosis median age was 68 years (range 37–88 years). 62 women were diagnosed with a tumor ≤ T2 (82.7%), 39 women presented with nodal involvement (52%) at time of first diagnosis and 22 at time of first recurrence (29.3%) (Table 1). All in all, 21 patients (28%) received radiotherapy to the vulva only, 18 patients (24%) received radiotherapy to the lymphatic drainage (inguinal and/or iliacal) only and 36 patients (48%) received radiotherapy to both vulva and lymphatic drainage. All patients received radiotherapy with curative intent with radiation doses > 45 Gy BED (biological effective dose, calculated with an alpha/beta of 10). Mean BED was 63.7 Gy (range 46.7–75.15 Gy). 72 patients received more than 50 Gy BED, 55 received more than 60 Gy BED, and 18 received more than 70 Gy BED (Table 2). Median follow-up of the entire cohort was 26.4 months (range 2.4–160.3 months, counted from the start of radiotherapy). 23 patients had locoregional relapse (30.7%), 7 developed systemic recurrence (9.3%), and 35 died (46.7%).

44 patients underwent radio-/radiochemotherapy as part of the initial treatment, 40 of them as adjuvant treatment after resection, three in a neoadjuvant setting and one as definitive treatment. 6 patients received concomitant chemotherapy [MitomycinC/5-FU (*n* = 5) or Cisplatin weekly (*n* = 1)]. After a median follow-up of 28.3 months (range 2.4–128.3 months) of this subgroup, 8 patients had locoregional relapse (18%), 5 patients developed systemic recurrence (11.4%), and 15 patients died (34%).

31 patients received radiotherapy because of disease recurrence, 27 of them as adjuvant treatment after resection, and 4 as definitive treatment. After a median follow-up of 14.4 months (range 2.5–160.3 months), 15 patients had locoregional relapse after radiotherapy (48.4%), two developed systemic recurrence (6.5%) and 20 had died (64.5%).

### Results of p16^INK4a^ Immunohistochemistry and HPV DNA Genotyping (Table 3)

Tissue specimen from time of primary diagnosis and/or tumor specimen from disease recurrence were available for analysis. For all 44 patients who underwent radio-/radiochemotherapy as part of the initial treatment tissue specimen from time of primary diagnosis were available for analysis. For all 31 patients who received radiotherapy because of disease recurrence, tissue specimen were available from diagnosis of recurrence. From this subgroup, additional tissue specimens from time of initial diagnosis were available for 13 patients. Interestingly, one patient initially tested positive for HPV DNA but without p16^INK4a^-overexpression did not show HPV DNA in tissue specimens of disease recurrence. Two women initially showing no p16^INK4a^-overexpression and no HPV DNA showed p16^INK4a^-overexpression at recurrence. For statistical analyses, HPV DNA- and p16^INK4a^-status at time of radiotherapy were used.

**Table 3:** Results of p16^INK4a^-immunostaining and expression of HPV DNA.

		**p16**^**INK4a**^		**Total**
		**Negative**	**Positive**	
HPV DNA	Negative	53 (70.67%)	6 (8%)	59 (78.67%)
	Positive	3 (4%)	13 (17.3%)^*^	16 (21.3%)
Total		56 (74.67%)	19 (25.3%)	75 (100%)

* *cHPPVC*.

59/75 (78.7%) of VSCC were tested HPV DNA-negative, whereas HR-HPV DNA was detected in 16/75 (21.3%) of the tumors. HPV 16 was the most frequently detected genotype (15/16, 93.75%), only one tumor specimen was positive for HPV 33/59. 19/75 patients (25.3%) showed p16^INK4a^-overexpression, defined as diffuse staining in more than 50% of tumor cells; 56/75 patients (74.67%) were tested p16^INK4a^-negative, showing only focal or no staining at all. 53/86 patients (70.67%) were both HPV DNA and p16^INK4a^-negative, while 13/75 (17.3%) were tested positive for both parameters. 6/75 patients (8%) were tested p16^INK4a^-positive without detection of HPV DNA. 3/75 patients (4%) were HPV DNA-positive without showing p16^INK4a^-overexpression (Table 3).

Chi-Square/Fisher exact test revealed significant correlation between detection of HPV DNA and p16^INK4a^-overexpression (*p* <0.001). Furthermore, a significant correlation between cHPPVC and tumor stage (*p* = 0.001) and p16^INK4a^ -status and tumor stage (*p* = 0.003) could be observed. Interestingly, cHPPVC and p16^INK4a^-overexpression were associated with higher tumor stage (>T2). Chi-Square/Fisher exact test revealed no further correlations of cHPPVC or p16^INK4a^-overexpression with any of the assessed pathological, patients' or treatment characteristics like age, date of primary diagnosis, nodal status, extracapsular tumor spread, grading, lymphovascular space invasion, radiation dose or setting of radiotherapy (adjuvant vs. definitive vs. neoadjuvant) (see also Tables 2, 3).

### Survival Endpoints for the Entire Cohort

Kaplan-Meier-estimated median PFS of the entire cohort was 28 months (95%-CI 0-77.4 months) with 2- and 5-year-PFS rates of 51.7 and 46.4%, respectively. Estimated median LC had not been reached at the time of analysis. 2- and 5-year LC rates were 69.2 and 64.1%, respectively. In the entire cohort, seven patients (9.3%) showed systemic recurrence. Kaplan-Meier estimated 1- and 2-year DC rates were 91.4 and 89.4%, respectively. All seven patients were in the non-cHPPVC group, so that further statistical analyses of DC were not reasonable. Estimated median OS was 66.4 months with 2-, 5-, and 10-year-OS rates of 58.9, 51.5, and 45.9%, respectively.

### Survival Endpoints by p16 ^INK4a^-Status Alone

**Figure 1:**
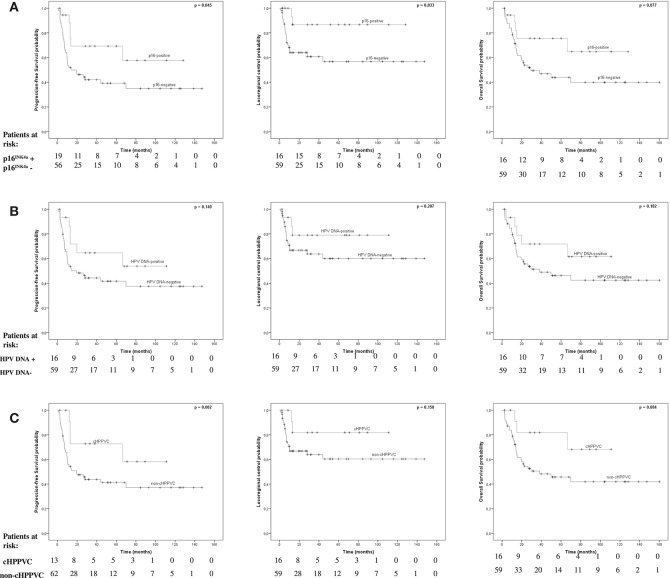
Survival endpoints by, p16^INK4a^-, HPV-, and cHPPVC-status. Kaplan-Meier estimated Progression-free survival (PFS), Locoregional Control (LC), and Overall Survival (OS) stratified by p16^INK4a^-status **(A)**, HPV DNA-status **(B)**, and cHPPVC-status **(C)** at time of radiotherapy.

p16^INK4a^-overexpression was associated with significantly better PFS and LC rates and a strong trend for a better OS (Figure 1). Kaplan-Meier-estimated median PFS, LC, and OS for p16^INK4a^-positive patients had not been reached at time of analysis. For p16^INK4a^-negative patients median PFS and OS were 14.1 months (95%-CI 0-32.9 months) and 29.3 months (95%-CI 0-61.6 months), respectively, estimated median LC had not been reached, yet. Patients with p16^INK4a^-overexpression showed significantly better PFS (*p* = 0.045) with 5-year-PFS rates of 69.3 vs. 39.2% for p16^INK4a^-negative patients. Patients with p16^INK4a^-overexpression showed significantly better LC rates (*p* = 0.033) with 5-year LC rates of 86.7 vs. 56.7% for p16^INK4a^-negative patients. p16^INK4a^-overexpression was associated with better, albeit not significant, OS (*p* = 0.077) with 5-year-OS rates of 75.6 vs. 43.9% for p16^INK4a^-negative patients.

### Survival Endpoints by HPV DNA-Status Alone

Detection of HPV DNA alone was not associated with significantly better PFS, LC or OS rates (Figure 1). Kaplan-Meier-estimated median PFS for HPV-DNA-positive patients had not been reached at time of analysis and was 19.4 months (95%-CI 0-39.7 months) for HPV-DNA-negative patients. There were no significant differences regarding PFS (*p* = 0.14) with 5-year-PFS rates of 64.6% for HPV DNA-positive patients vs. 41.7% for HPV DNA-negative patients. Estimated median LC for both HPV DNA-positive and -negative patients had not been reached at time of analysis. There were no significant differences regarding LC (*p* = 0.207) with 5-year-LC rates of 79% for HPV DNA-positive patients vs. 60% for HPV DNA-negative patients. Estimated median OS for HPV DNA-positive patients had also not been reached yet, and was 38.3 months for HPV DNA-negative patients (95%-CI 0-87.2 months). There were no significant differences regarding OS (*p* = 0.182) with 5-year-OS rates of 71.8% for HPV DNA-positive patients vs. 46.3% for HPV DNA-negative patients.

### Survival Endpoints by cHPPVC-Status

cHPPVC-status was associated with a strong trend for better PFS and OS rates, with no significant differences in LC rates (Figure 1). Kaplan-Meier-estimated median PFS for cHPPVC patients had not been reached at time of analysis and was 19.4 months (95%-CI 1.4-37.4 months) for non-cHPPVC patients. Patients with cHPPVC showed a strong trend for better PFS (*p* = 0.082) with 5-year-PFS rates of 72.7 vs. 41.3% for non-HPPVC patients. Estimated median LC for both cHPPVC and non-cHPPVC patients had not been reached at time of analysis. There were no significant differences regarding LC (*p* = 0.158) for patients with cHPPVC compared to non-cHPPVC patients with 5-y-LC rates of 81.8 vs. 60.4%. Estimated median OS for cHPPVC patients had also not been reached yet, and was 38.3 months for non-HPPVC patients (95%-CI 0-82.4 months). Patients with cHPPVC showed a strong trend for better OS (*p* = 0.084) with 5-year-OS rates of 81.1 vs. 45.7% for non-HPPVC patients.

## Discussion

Data on the prognostic significance of HPV-driven carcinogenesis in vulvar cancer are discussed controversially. The main challenge in comparing literature are differences in defining HPV-driven carcinogenesis. Biologically, three criteria are necessary to prove a causative role of HPV in carcinogenesis: presence of HPV DNA, transcription and translation of the E6 and E7 oncogenes and dependence of HPV-transformed cells on their continuous expression (6). Due to difficulties in defining such criteria in daily clinical practice, practicable markers indicating a functional, transforming relevance of HPV in tumor cells are needed. In this context, p16^INK4a^ has become a widely used biomarker for HPV-transformed cells (6, 16), e.g., in the anogenital and head and neck region (6, 10–12).

In our cohort, concomitant p16^INK4a^-overexpression and detection of HPV DNA was associated with a trend for better PFS and OS, whereas p16^INK4a^-overexpression alone was associated with significantly better PFS and LC and a strong trend for a better OS. Sole detection of HPV DNA was not associated with significantly better PFS, LC, or OS rates.

Several studies were published assessing the prognostic role of HPV-driven carcinogenesis in vulvar cancer, most of them using either p16^INK4a^-overexpression (17–20) or HPV DNA (17, 18, 21–23) as marker for HPV-driven carcinogenesis. They couldn't prove an independent prognostic role of HPV DNA in vulvar cancer (21, 22) or solely claimed a trend for better survival (23). Interestingly, in studies using p16^INK4a^-overexpression only as surrogate marker, a survival benefit has been reported (17–20). A recently published study describes a survival benefit for HPV-driven VSCC patients undergoing primary resection (24). VSCCs were considered HPV-related in case of either >25% p16^INK4a^-expression and HPV-positivity or >25% p16^INK4a^-expression and high grade squamous intraepithelial lesion next to the tumor without HPV-positivity (24). It has been shown that precursor lesions of vulvar cancer are more often HPV-associated than invasive VSCCs (>80 vs. 25%) (1), so that HPV-driven etiology might be overestimated in that collective. Additionally, HPV-negative patients presented with positive lymph nodes more frequently, being strongly associated with a worse prognosis which might bias the reported results.

Our study reports one of the largest cohorts of irradiated VSCC patients with assessed HPV DNA and p16^INK4a^ status. An outstanding characteristic of our study is that concomitant detection of HPV DNA and p16^INK4a^-overexpression was regarded mandatory for indicating HPV-driven carcinogenesis (7). As all patients required radiotherapy or already were in the situation of recurrence this is considered to be a negatively selected collective. Furthermore, data might be biased due to the retrospective nature of the study. As only 17.3% were identified having cHPPVC, the cohort might be too small to detect any statistically significant differences in oncological outcome. However, Alonso et al. also used HPV DNA in combination with p16^INK4a^-overexpression as marker for HPV-driven carcinogenesis. Only cases with diffuse staining, defined as continuous staining of cells of the basal and parabasal layers, were considered p16^INK4a^-positive. The authors also couldn't find significant differences in outcome for HPV-driven VSCC patients (25), what is consistent with our data. There were no anaylses conducted regarding the prognostic significance of p16^INK4a^-overexpression alone, what was associated with significantly better LC, PFS, and better OS in our cohort, irrespective of HPV DNA-detection.

Recently, more and more evidence arises revealing a mismatch between p16^INK4a^-overexpression and HPV DNA-detection in VSCCs. In our cohort, six patients were tested p16^INK4a^-positive but HPV DNA-negative, three were HPV DNA-positive but p16^INK4a^-negative. A large cohort study reported only 87.9% to be both HPV DNA- and p16^INK4a^-positive (1). Sznurkowsky et al. (15) described 29% of 85 tumors p16^INK4a^-positive but HPV DNA-negative and 24% p16^INK4a^-negative but HPV DNA-positive. Only p16^INK4a^-overexpression correlated with prolonged OS and predicted a better response to irradiation (15). Additionally, a large meta-analysis of 2,309 patients suggested that p16^INK4a^-status itself is associated with a better prognosis in vulvar cancer patients and indicates higher radiosensitivity (14). There are current studies investigating other molecular mechanisms leading to higher radiosensitivity of p16^INK4a^-positive VSCCs, suggesting that immunologic effects depending on p16^INK4a^-overexpression contribute to better outcome (26).

Patients with cHPPVC, indicating an etiological relevance of HPV in the respective tumors, showed a better, albeit not significant, prognosis. Due to the rather low incidence of cHPPVC, the sample size might be too small for showing any statistically significant differences in our cohort. Our observations should consequently be validated in larger patient cohorts. Interestingly, p16^INK4a^ overexpression alone seems to be a prognostic factor for survival in vulvar cancer and indicates better prognosis after radiotherapy, independent of detection of HPV DNA. p16^INK4a^ should be used as surrogate marker for HPV-driven carcinogenesis in vulvar cancer only with caution, as more and more evidence arises that there seem to be other HPV-independent mechanisms in vulvar cancer leading to p16^INK4a^ overexpression. Further analyses are necessary investigating potential molecular mechanisms for p16^INK4a^ overexpression in vulvar cancer.

## Data Availability

The datasets for this manuscript are not publicly available due to privacy and data protection reasons of the patient data included in the analysis. Requests to access the datasets should be directed to NA, nathalie.arians@med.uni-heidelberg.de.

## Ethics Statement

This study was carried out in accordance with the recommendations of the Declaration of Helsinki and institutional ethical policies of the University Hospital Heidelberg. The protocol was approved by the ethics committee of the University Hospital Heidelberg (S-308/2012, 24.09.2012, Amendment 04.06.2018). As the data were analyzed retrospectively and anonymously and treatment of patients was not affected by this study, no written informed consent from each individual patient was necessary according to institutional standards and the local ethics committee decision.

## Author Contributions

NA, E-SP, MR, and KL: conceptualization. NA and TN: data curation and investigation. NA: formal analysis and writing—original draft. NA, E-SP, and MR: methodology and validation. JD, MK, and KL: project administration, resources, and supervision. E-SP and SK: writing—review and editing. All authors contributed substantially to the work reported.

### Conflict of Interest Statement

The authors declare that the research was conducted in the absence of any commercial or financial relationships that could be construed as a potential conflict of interest.
